# An ultra-minimally invasive attempt: endoscopic hand-suturing for delayed anastomotic fistula after low rectal cancer surgery

**DOI:** 10.1055/a-2886-5775

**Published:** 2026-06-30

**Authors:** Lizhou Dou, Angshu Cai, Bowen Zha, Guiqi Wang, Shun He

**Affiliations:** 1 Department of EndoscopyNational Cancer Center/National Clinical Research Center for Cancer/Cancer Hospital, Chinese Academy of Medical Sciences and Peking Union Medical CollegeBeijingChina

## A case description



**Video 1** Endoscopic hand-suturing for closure of a delayed anastomotic fistula after ultra-low anterior resection.


A delayed anastomotic fistula after low rectal cancer surgery is defined as a defect occurring more than 30 days postoperatively, forming a fistulous communication with adjacent structures. 1​ ​2​ Anastomotic fistulas are associated with higher rates of permanent stoma and functional impairment 2​ ​3​ and can significantly reduce patients’ quality of life. 4​ We report a novel endoscopic hand-suturing (EHS) technique for managing delayed anastomotic fistulas after low rectal cancer surgery.

**Fig. 1:**
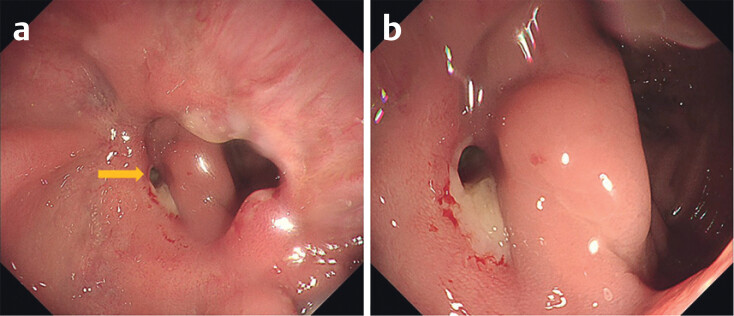
Delayed anastomotic fistulas after ultra-low anterior resection: ( **a** ) an endoscopic view showing a mucosal defect (arrow) around the anastomosis; ( **b** ) the fistula with purulent discharge, surrounded by erythematous and edematous mucosa.

**Fig. 2:**
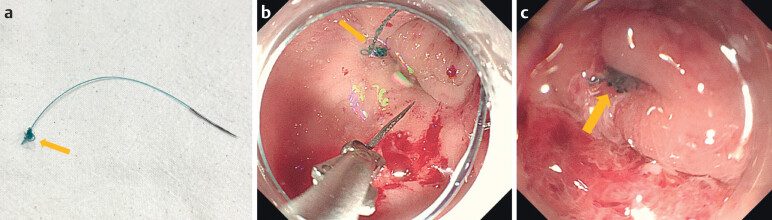
An endoscopic hand-suturing device: ( **a** ) absorbable barbed suture with a needle (VLOCL0803; Covidien, Mansfield, MA, USA) used for endoscopic suturing, with the suture tail knotted (arrow); ( **b** ) endoscopic suturing using the custom-designed needle holder to manipulate the suture (entrusted manufacturer: Vedkang, Jiangsu, China); ( **c** ) the knot at the suture tail (arrow) secures the suture during tightening, preventing slippage.

**Fig. 3:**
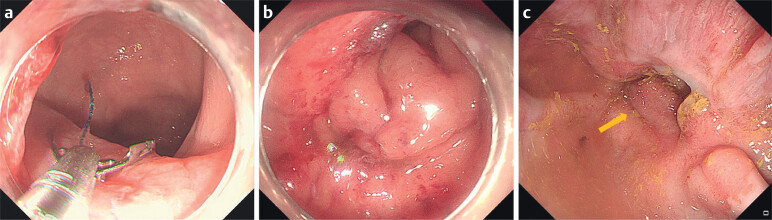
Endoscopic hand-suturing closure of the fistula: ( **a** ) after completing suturing and tightening the suture, the remaining suture tail was secured with a titanium clip. ( **b** ) The fistula was completely closed after suturing. ( **c** ) Follow-up colonoscopy at 3 months showed healed fistula and spontaneous detachment of the titanium clip.

A 60-year-old man underwent ultra-low anterior resection (ULAR) for a malignant rectal tumour located 3 cm from the anal verge. The postoperative TNM stage was T1N0M0, and no adjuvant therapy was administered. The perioperative course was uneventful, and the diverting ileostomy was reversed as scheduled. One year after surgery, the patient presented with low-grade fever, localized lower abdominal pain, and tenesmus. Imaging and endoscopic evaluation ( Fig. 1 ) revealed a mucosal defect measuring 1.0×0.6 cm at the anastomosis, consistent with a delayed anastomotic fistula without urethral involvement. After being fully informed of the condition and associated risks, the patient elected to undergo EHS in combination with antibio-tic therapy. Under endoscopic guidance, purulent secretions and necrotic tissue around the fistula were debrided, followed by suturing using our previously reported original suturing device. 5​ The suture was secured to the mucosa by knotting the tail end, allowing fixation during tightening ( Fig. 2 ). After continuous suturing, the suture was tightened and fixed with titanium clips, achieving complete fistula closure ( Fig. 3a, b , Video 1 ). The patient was discharged on postoperative day 2 without adverse events. Follow-up colonoscopy at 3 months confirmed complete fistula closure ( Fig. 3c ), and the patient remained asymptomatic.

Endoscopic management of postoperative rectal anastomotic fistulas remains exploratory. This case demonstrates the safety and feasibility of EHS as a novel treatment option for delayed anastomotic fistulas following ULAR.

Endoscopy_UCTN_Code_TTT_1AQ_2AG

## References

[1] Shimomura M Yoshimitsu M Tsukada Y Late anastomotic complication after laparoscopic surgery for clinical stage I low rectal cancers located within 5 cm of the anal verge: Sub-analysis of the ultimate trial Ann Gastroenterol Surg 2025 9 719 729 40607283 10.1002/ags3.12904 PMC12211094

[2] Lim S B Yu C S Kim C W Late anastomotic leakage after low anterior resection in rectal cancer patients: clinical characteristics and predisposing factors Colorectal Dis 2016 18 O135 O140 26888300 10.1111/codi.13300

[3] Hain E Manceau G Maggiori L Bowel dysfunction after anastomotic leakage in laparoscopic sphincter-saving operative intervention for rectal cancer: A case-matched study in 46 patients using the Low Anterior Resection Score Surgery 2017 161 1028 1039 27894710 10.1016/j.surg.2016.09.037

[4] Ashburn J H Stocchi L Kiran R P Consequences of anastomotic leak after restorative proctectomy for cancer: effect on long-term function and quality of life Dis Colon Rectum 2013 56 275 280 23392139 10.1097/DCR.0b013e318277e8a5

[5] Song S Zhang C Wang G Endoscopic submucosal dissection combined with endoscopic hand-suturing for a superficial esophageal cancer in a diverticulum Endoscopy 2024 56 E772 E773 39242098 10.1055/a-2381-4993 PMC11379532

